# Family-Based Digital Lifestyle Intervention for Hispanic Adolescents and Their Parents: Iterative Co-Design and Development Study

**DOI:** 10.2196/73848

**Published:** 2026-02-05

**Authors:** Sara Mijares St George, Blanca S Noriega Esquives, Rafael Leite, Vanina Pavia Aubry, Rana Saber, Yaray Agosto, Marissa Kobayashi, Guillermo Prado

**Affiliations:** 1 Department of Public Health Sciences, University of Miami Miller School of Medicine, 1120 NW 14 Street, Suite 1009, Miami, FL, 33136, United States, 1 305 243 0726; 2 Sylvester Comprehensive Cancer Center, University of Miami Miller School of Medicine, Miami, FL, United States; 3 Department of Psychiatry and Human Behavior, Alpert Medical School of Brown University, Providence, RI, United States; 4 Weight Control and Diabetes Research Center, The Miriam Hospital, Providence, RI, United States; 5 Valorous Health Innovation, LLC, Seattle, WA, United States; 6 Department of Medical Social Sciences, Feinberg School of Medicine, Northwestern University, Chicago, IL, United States; 7 Department of Pediatrics, University of Miami Miller School of Medicine, Miami, FL, United States; 8 Department of Psychology, University of Miami, Coral Gables, FL, United States; 9 School of Nursing and Health Studies, University of Miami, Coral Gables, FL, United States

**Keywords:** digital health, family-based, obesity prevention, Hispanic, adolescent, user-centered design

## Abstract

**Background:**

Hispanic youth in the United States have the highest rates of pediatric obesity and do not often meet national guidelines for physical activity and dietary intake. Family-based interventions can improve health outcomes in both youth and their parents and are highly relevant to Hispanics due to the cultural value of familismo (familism). However, few existing family-based obesity prevention interventions for Hispanics target adolescents and their parents, and those that do are not designed to facilitate widespread reach.

**Objective:**

This study describes the development of Healthy Juntos (Healthy Together), a family-based intervention for Hispanic adolescents and their parents that leverages the web and smartphone technology to prevent the onset of adolescent obesity by promoting healthy lifestyle behaviors (physical activity and diet).

**Methods:**

We used an iterative co-design process guided by the Integrate, Design, Assess, and Share (IDEAS) framework, which outlines 10 phases for developing digital interventions. Hispanic adolescents at risk for obesity and their parents (n=90; 45 dyads) participated across different phases of the intervention development process. We conducted qualitative interviews to understand their needs and preferences and to gather feedback on a series of intervention prototypes (conceptual, paper and minimally functional, and fully functional).

**Results:**

Participants reported using technology for their health in limited ways (eg, to search for medical symptoms and recipes). They described the importance of having interactive and social features as part of a family-based digital health intervention. Their suggestions related to content, functionality, and aesthetics resulted in a fully functional prototype of a digital lifestyle intervention for Hispanic adolescents and their parents.

**Conclusions:**

The iterative co-design process was crucial for refining the Healthy Juntos intervention. Our next steps are to evaluate its feasibility, acceptability, and preliminary effects through a pilot randomized controlled trial.

## Introduction

Obesity disproportionately affects Hispanics in the United States, with Hispanic youth experiencing the highest prevalence (26%) relative to non-Hispanic Black (24%), White (16%), and Asian (9%) youth [1]. This disparity continues into adulthood, where the prevalence of overweight or obesity is higher for Hispanic men (88%) and women (79%) compared to non-Hispanic White men (75%) and women (66%) [2,3]. Furthermore, Hispanic adolescents exhibit a greater increase in BMI from ages 12 to 19 years compared to their non-Hispanic White peers [4,5]. Likely contributing to these rates, most Hispanic youth and adults do not meet national guidelines for physical activity or diet [6-9]. Hispanic youth engage in only 35 of the recommended 60 minutes per day of moderate-to-vigorous physical activity (MVPA) and are sedentary for ~10 hours daily [6]. They also show poor adherence to US dietary guidelines, as indicated by a low mean Healthy Eating Index (HEI) score of 53.8 out of 100, including low scores for whole fruit (2.1 out of 5) and total vegetable intake (2.0 out of 5) [7]. With Hispanics comprising 62.5 million Americans and projected to represent nearly a quarter of the US population by 2040 [10,11], the delivery of culturally relevant, evidence-based, and scalable obesity prevention interventions in this population is a major public health priority.

Adolescence represents a critical window for obesity prevention efforts. Although youth with obesity are 5 times more likely to have obesity in adulthood, this pattern is driven largely by adolescents, an estimated 70% of whom continue to have obesity after age 30 years [12]. We conducted a comprehensive systematic review and meta-analysis of pediatric obesity prevention and treatment interventions among Hispanic youth aged 0‐18 years [13]. Our quantitative synthesis of 105 interventions published between 2000 and 2020 showed small but significant overall effects on youth weight status (Hedges *g*=−0.15), waist circumference (Hedges *g*=–0.15), physical activity (Hedges *g*=0.12), fruit and vegetable intake (Hedges *g*=0.08), and sugar-sweetened beverage intake (Hedges *g*=–0.07). Importantly, interventions targeting early-to-middle adolescents (aged 12‐15 years) showed significantly larger effects on weight status (Hedges *g*=−0.27) compared to those conducted during early childhood (Hedges *g*=−0.13), childhood (Hedges *g*=−0.03), and late adolescence (Hedges *g*=−0.09), underscoring the importance of this developmental stage.

During early to middle adolescence, although adolescents begin to seek independence, parents still exert significant influence on eating habits (eg, overseeing grocery shopping, determining the frequency of family meals, and establishing normative expectations), as well as on household physical activity patterns [14,15]. Because Hispanic parents prioritize familismo (familism), or family interdependence and loyalty, and generally have later age expectations for youth to engage in independent behaviors than other ethnic groups [16,17], family-based approaches (ie, those that actively involve family members in the behavior change process) remain highly culturally relevant throughout adolescence. Interventions in our meta-analysis involving the family had significant effects on Hispanic youth’s weight status (Hedges *g*=−0.19). Despite this, only 6 of the 25 interventions for early-to-middle adolescents were primarily family-based, reinforcing the need for more family-based obesity prevention efforts among Hispanic adolescents [18].

Digital technology offers a promising avenue for delivering family-based lifestyle interventions to Hispanic adolescents and their parents. While digital interventions have been shown to improve adolescent weight [19] and lifestyle behaviors [20,21], especially when involving parents [19,22,23], their application among ethnic minority youth and their families remains limited [24]. In our aforementioned meta-analysis, for example, only 4% of studies leveraged some form of technology (eg, the internet, SMS text messaging) [13]. Thus, there is a clear opportunity to increase digital lifestyle intervention research among Hispanics, who are just as likely as non-Hispanic Whites to own smartphones (93% vs 91%) and more likely (20% vs 12%) to be smartphone-only internet users [25]. Hispanic adolescents aged 13‐17 years also report high access to smartphones (96%) and are more likely than their non-Hispanic White peers to report using the internet “almost constantly” (55% vs 38%) [26]. By capitalizing on the widespread adoption of digital technologies, researchers should prioritize the development and evaluation of digital interventions that can empower Hispanic youth and their families to adopt healthier lifestyles. As such, the purpose of this study is to describe the development of a family-based digital intervention, Healthy Juntos (Healthy Together), for promoting healthy lifestyle behaviors (physical activity and diet) in Hispanic adolescents. We systematically report how user-centered qualitative and quantitative feedback from Hispanic parents and adolescents was integrated to refine the intervention’s content, functionality, and aesthetic design, resulting in a fully functional prototype ready for evaluation in a pilot randomized controlled trial (RCT).

## Methods

### Overview

We used the Integrate, Design, Assess, and Share (IDEAS) framework to guide our intervention development and iterative co-design process [27]. The IDEAS framework outlines a systematic set of 10 phases for developing and evaluating digital health interventions, which include (1) empathizing with target users, (2) specifying target behavior(s), (3) grounding intervention development in behavioral theory, (4) brainstorming implementation strategies, (5) prototyping potential products, (6) gathering user feedback, (7) building a minimum viable product, (8) pilot testing, (9) evaluating efficacy, and (10) sharing findings. While these phases are outlined sequentially, this framework encourages a flexible and iterative approach, allowing developers to revisit and refine different phases as needed. This initial report outlines our application of the first 7 phases of the IDEAS framework (Table 1).

**Table 1.** Application of the IDEAS framework to the development of Healthy Juntos.

IDEAS framework		Healthy Juntos
Empathize with users		Interviews and focus groups using conceptual prototypes (n=20 dyads)
Specify target behaviors		Moderate-to-vigorous physical activity (MVPA), diet quality, sedentary behavior, BMI
Ground in theory		Family systems with social cognitive and self-determination theories
Brainstorm		Development of paper and minimally functional prototypes
Prototype potential products		Development of paper and minimally functional prototypes
Gather user feedback		Evaluation of paper and minimally functional prototypes (n=15 dyads)
Build a minimum viable product		Development and evaluation of fully functional prototype (field trial; n=10 dyads)
Pilot test		Next steps
Evaluate efficacy		Next steps
Share findings		Next steps

**Figure 1.**
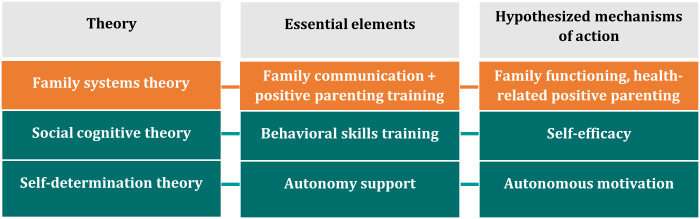
Conceptual framework.

During phase 1 of the IDEAS framework, we collected qualitative data from Hispanic parents and their 12‐15-year-old adolescents to better understand their lifestyle behaviors and use of technology for health and to gather their feedback on initial intervention concepts and prototypes (“conceptual prototypes”). During phases 2-3, we specified our target behaviors (ie, increasing MVPA, improving dietary intake, reducing sedentary behavior) and grounded our intervention development in behavioral and motivational theories. Given recommendations to integrate family systems theory [28] with social cognitive [29] and self-determination theories [30] to develop interventions for addressing obesity in high-risk youth [31], our conceptual framework integrated family communication and positive parenting with behavioral strategies (eg, goal setting) and elements involved in fostering autonomous motivation (eg, autonomy support; Figure 1). We additionally considered Hispanic cultural values and constructs, including familismo (familism), confianza (trust), personalismo (personal connection), and acculturation (ie, the process by which individuals from one culture adapt to the behaviors, attitudes, and values of another culture) [32] in our conceptualization of the intervention. During phases 4-5, we designed initial intervention prototypes (“paper and minimally functional prototypes”). In phase 6, we gathered qualitative feedback on these prototypes from Hispanic parent-adolescent dyads, which informed the development of a “fully functional” prototype. The fully functional prototype was then assessed in phase 7 through a small-scale field trial.

### Participants

A total of 45 different sets of Hispanic-parent adolescent dyads participated across the 7 phases of the intervention development process.

#### Eligibility Criteria

Families were eligible to participate if (1) the adolescent’s primary caregiver self-identified as Hispanic; (2) the adolescent was between the age of 12 and 15 years; (3) the adolescent lived with an adult primary caregiver (ie, biological parent or legal guardian; herein referred to as “parent”) willing to participate; (4) both parent and adolescent owned a smartphone with internet access; (5) the adolescent did not meet recommendations for fruit and vegetable intake as determined by the fruit and vegetable brief measure [33]; (6) the adolescent did not meet physical activity guidelines as determined by the 60-minute MVPA screening measure [34]; and (7) the adolescent self-reported engaging in more than 2 hours of daily screen time, based on three items from the Youth Risk Behavior Surveillance Survey [35].

Families were excluded if (1) the adolescent’s BMI-for-age-and-gender was <5% (underweight) or ≥ 95% (obesity); (2) the adolescent had a chronic medical condition (eg, type 2 diabetes) that required intensive lifestyle modification; (3) the adolescent had a diagnosed developmental delay that would interfere with understanding program materials; (4) the parent or adolescent had a diagnosed medical or psychiatric condition and was taking medications that would interfere with changes to physical activity or diet (eg, adolescent was diagnosed with Attention Deficit Hyperactivity Disorder and was on stimulant medication); and (5) the family was planning to move out of South Florida during the study period.

#### Recruitment

We used several recruitment strategies, including partnering with local pediatric primary care clinics in South Florida, drawing from consent to contact lists, offering monetary incentives for referrals from enrolled study participants, advertising the study on social media, and distributing flyers at community events.

Our research team contacted a total of 540 potential families. Of those, 45% were ineligible, 31% did not respond to our calls, 12% refused to participate, and 2% were enrolled in a competing study (see Multimedia Appendix 1 for participant flow diagram). Of the 55 eligible families, 82% (n=45 families) provided written informed consent and assent and agreed to participate in the study.

### Procedures

#### Overview

We prescreened potential participants in person at pediatric primary care clinics or by phone. To confirm the adolescent’s eligibility, study staff measured their height and weight and calculated their BMI percentile. Due to the COVID-19 pandemic and the university policy to conduct research remotely at the time, we conducted recruitment for our field trial (IDEAS phase 7) exclusively by phone and determined adolescent eligibility using parent-reported height and weight to estimate adolescents’ BMI percentile.

We audio- or video-recorded all qualitative data collection sessions, and they were transcribed verbatim by a professional transcription agency (National Captioning Institute). Transcription was performed singly, and a member of our research team conducted quality checks of the transcripts against the original audio recordings to ensure completeness and accuracy. If participants did not consent to being recorded, extensive notes were taken instead.

Different sets of Hispanic parent-adolescent dyads participated across 3 phases of data collection (conceptual prototypes, n=20 dyads; paper and minimally functional prototypes, n=15 dyads; and fully functional prototype, n=10 dyads). Procedures for each of these phases are detailed below.

#### Conceptual Prototypes

Parents and their adolescents (n=20 dyads) participated in separate focus groups or individual interviews. We initially set out to conduct focus groups; however, due to scheduling challenges, we used a combination of group-based and individual interview approaches. The purpose of these initial focus groups or interviews was to understand the needs and behaviors of our target users. Prior to qualitative data collection, each participant completed a brief demographic survey. Parents self-reported their age, sex, country of birth, years living in the United States, marital status, education, employment, household income, and potential history of diabetes. Adolescents self-reported their age, sex, country of birth, and years of living in the United States. Parents and adolescents also completed the Mobile Device Proficiency Questionnaire (MDPQ-16) [36], a 16-item validated instrument that assesses proficiency related to mobile device use. Sample items include “I can find health information online,” “I can listen to music,” and “I can send emails” (all using a mobile device). Participants rated their level of proficiency on a 5-point response scale ranging from never tried at all to very easily. The measure yields 8 subscales (eg, communication, entertainment) and one total score, with higher scores reflecting greater mobile device proficiency.

We used a semistructured guide (Multimedia Appendix 2) with open-ended questions designed to understand participants’ current lifestyle behaviors (eg, “How [if at all] are you healthy together with your parent/adolescent?”) and use of technology for health (eg, “How [if at all] have you used technology for your health/health care?”). We additionally gathered participants’ feedback on initial intervention concepts and prototypes (“conceptual prototypes”). Bilingual study staff conducted focus groups and interviews in participants’ homes or at the University of Miami in either English (one adolescent focus group [n=3], n=8 individual adolescent interviews) or Spanish (one adolescent focus group [n=2], n=7 individual adolescent interviews; all parent focus groups and individual interviews). Focus group sessions were ~55 minutes, and individual interviews were ~36 minutes. Participating parents and their adolescents received US $50 and 2 movie tickets, respectively.

#### Paper and Minimally Functional Prototypes

Based on feedback from our initial focus groups and interviews, our team developed a series of paper and minimally functional prototypes, or those that meet some, but not all, specifications for a product. Our paper prototypes consisted of physical printouts of illustrations depicting intervention features. Our minimally functional prototypes consisted of a webpage accessed via smartphone with learning activities that aligned with didactic content.

A different set of parents and adolescents (n=15 dyads) completed the aforementioned demographic survey and participated in separate individual interviews. We used a semistructured guide (Multimedia Appendix 3) with open-ended questions designed to solicit participants’ feedback on potential logos (eg, “What images come to mind when you hear ‘Healthy Juntos’ [Healthy Together]”) and to encourage participants to “think aloud” while interacting with the paper and minimally functioning prototypes to understand their navigation of the intervention, identify potential problems, and assess their interpretation of the prototype’s design elements. We also collected feedback on participants’ favorite and least favorite components, suggested improvements for the functionality of game prototypes, and impressions of the overall design of the program. Bilingual study staff conducted interviews in participants’ homes or a public setting (eg, library) in either English (n=13 adolescents, n=3 parents) or Spanish (n=2 adolescents; n=12 parents). The parent and adolescent interviews lasted ~42 minutes and ~30 minutes, respectively.

After the interviews, participants completed the Usefulness, Satisfaction, and Ease of use (USE) questionnaire [37]. This 30-item measure assesses four dimensions of usability: usefulness (8 items), ease of use (11 items), ease of learning (4 items), and satisfaction (7 items). Response options ranged from 1 (strongly disagree) to 7 (strongly agree). The 4 mean scores were averaged to calculate a total usability score, with higher scores indicating greater usability and satisfaction. Participating parents and their adolescents received US $60 and 3 movie tickets, respectively.

#### Fully Functional Prototype

Based on feedback from the paper and minimally functional prototypes, our team developed a fully functional prototype of a web-based application, available in English and Spanish, for an 8-week family-based intervention. Although ≥26 contact hours over 2‐12 months are recommended for pediatric obesity treatment [38], no equivalent recommendation exists for preventive interventions, with prior meta-analyses (including our own) finding no clear relationship between intended dose and weight outcomes [13,39]. The initial intervention duration was informed by the principal investigator’s (PI) prior work developing and evaluating an 8-week family-based (face-to-face) intervention that demonstrated postintervention improvements in the lifestyle behaviors of non-Hispanic Black adolescents and parents [40,41].

We evaluated our fully functional prototype in a field trial (n=10 dyads). Field trials involve the collection of data in users’ natural environments and evaluate technology for flaws that will only emerge through use over time [42]. We gave parents and adolescents a physical activity monitor (Fitbit) to track their steps and held a one-hour videoconference call to set up their usernames, sync their Fitbit devices to the app, and introduce them to our intervention. At the end of each week, participants rated the week’s content and provided feedback on what they liked, disliked, and would change about each session, as well as any technical issues they encountered. Participating families also received a follow-up phone call after Week 4 to check on their progress. At the end of the eight weeks, study staff invited families to participate in exit interviews via videoconference using a semistructured guide with open-ended questions (Multimedia Appendix 4). The interview guide was designed to capture participants’ experience using the program (eg, “How [if at all] did the program help you meet your goals?” and “How [if at all] did the program impact your relationship with your adolescent/parent?”). Bilingual study staff conducted interviews in either English (n=7 adolescents, n=1 parent) or Spanish (n=1 adolescent; n=7 parents). Interviews lasted an hour on average. Parents received a US $5 digital gift card for every weekly survey and a US $40 digital gift card for participating in the exit interview. Adolescents received a US $30 digital gift card for participating in the exit interview. Parents and adolescents were allowed to keep their Fitbit devices once the study was completed.

### Data Analysis

#### Quantitative Data

We summarized quantitative data using descriptive statistics and did not perform any inferential statistical tests. Analyses were conducted in SAS (version 9.4; SAS Institute Inc).

#### Qualitative Data

We used a rapid qualitative analysis to analyze qualitative data [43-45]. Rapid qualitative analysis leverages a team-based approach to summarize key points from qualitative data into a matrix, allowing for efficient and systematic exploration of relevant themes. This method can facilitate the development of technology-based interventions [46]. At each phase of qualitative data collection, we first reviewed transcripts for accuracy against the original audio or video recordings. We then developed a summary template to condense data from each transcript across a series of domains generated from the questions in the interview guides. Study staff created matrices that displayed all participant responses corresponding to each domain. In the matrices, each focus group or interview was represented by a unique row, and each domain was represented by a unique column. We distributed our completed matrices to study staff for revision and used a team-based discussion approach to draw key conclusions that informed our intervention development.

### Ethical Considerations

This study was approved by the institutional review board of the University of Miami (protocol ID number 20160415). Our team obtained written informed consent and assent from all participants in person or via video conference prior to data collection. We securely stored participant data on a protected, access-controlled University server.

## Results

### Participant Characteristics

A total of 45 Hispanic adolescents, identified as at-risk for obesity due to not meeting national recommendations for fruit and vegetable intake, physical activity, and sedentary behavior, and their parents participated in the development and co-design of Healthy Juntos (Table 2). Adolescents had an average age of 13.8 (1.2) years and a BMI percentile of 67.9 (23.7). They were predominantly female (27/45, 60%), US-born (23/45, 51%), and preferred English as their primary language (30/45, 67%). Parents had an average age of 44.3 (5.7) years and a BMI of 29.3 (8.6). They were predominantly female (42/45, 93%), married or living with someone (34/45, 73%), born outside the United States (43/45, 96%), had lived in the United States for more than 10 years (23/45, 51%), preferred Spanish as their primary language (40/45, 89%), and reported an annual household income below US $50,000 (41/45, 91%).

**Table 2.** Participant characteristics.

Characteristic	Total (n=45 adolescents and parents)	Conceptual prototype (n=20 adolescents and parents)	Paper and minimally functional prototype (n=15 adolescents and parents)	Fully functional prototype (n=10 adolescents and parents)
Adolescents				
Age (years), mean (SD)	13.8 (1.2)	13.5 (1)	14.2 (1.3)	13.9 (1.3)
BMI (%), mean (SD)	67.9 (23.7)	59.1 (26.1)	81.3 (10.5)	65.3 (25.5)
Sex (female), n (%)	27 (60)	13 (65)	9 (60)	5 (50)
Country of birth, n (%)				
United States	23 (51.1)	7 (35)	10 (66.7)	6 (60)
Venezuela	9 (20)	5 (25)	2 (13.3)	2 (20)
Cuba	5 (11.1)	3 (15)	1 (6.7)	1 (10)
Other	8 (17.8)	5 (25)	2 (13.3)	1 (10)
Years living in the United States, n (%)				
Less than 1 year	5 (11.1)	5 (25)	—	—
1‐9 years	18 (40)	8 (40)	4 (26.7)	6 (60)
More than 9 years	22 (48.9)	7 (35)	11 (73.3)	4 (40)
Spanish as preferred language, n (%)	15 (33.3)	8 (40)	4 (26.7)	3 (30)
Parents				
Age (years), mean (SD)	44.3 (5.7)	43.2 (6.2)	46.27 (4.7)	43.70 (5.6)
BMI, mean (SD)	29.3 (8.6)	30.1 (12.2)	30.36 (3.8)	25.83 (2.5)
Sex (female), n (%)	42 (93.3)	18 (90)	14 (93.3)	10 (100)
Relationship with adolescent				
Biological parent	44 (97.8)	19 (95)	15 (100)	10 (100)
Legal guardian	1 (2.2)	1 (5)	—	—
Country of birth, n (%)				
Venezuela	13 (28.9)	6 (30)	4 (26.7)	3 (30)
Cuba	8 (17.8)	4 (20)	3 (20)	1 (10)
United States	2 (4.4)	—	1 (6.6)	1 (10)
Other	22 (48.9)	10 (50)	7 (46.7)	5 (50)
Years living in the United States, n (%)				
Less than 3 years	10 (22.2)	9 (45)	—	1 (10)
3‐10 years	12 (26.7)	4 (20)	3 (20)	5 (50)
More than 10 years	23 (51.1)	7 (35)	12 (80)	4 (40)
Spanish as preferred language, n (%)	40 (88.9)	19 (95)	12 (80)	9 (90)
Marital status, n (%)				
Married or living with someone	34 (75.6)	15 (75)	12 (80)	7 (70)
Divorced or separated or widowed	9 (20)	3 (15)	3 (20)	3 (30)
Single	2 (4.4)	2 (10)	—	—
Education, n (%)				
Up to high school	12 (26.7)	5 (25)	3 (20)	4 (40)
Some college or college degree	26 (57.8)	13 (65)	11 (73.3)	2 (20)
Graduate or professional	7 (15.6)	2 (10)	1 (6.7)	4 (40)
Work status, n (%)				
Working full-time or part-time	29 (64.5)	10 (50)	11 (73.3)	8 (80)
Homemaker	5 (11.1)	4 (20)	1 (6.7)	—
Working in temporary jobs	5 (11.1)	3 (15)	1 (6.7)	1 (10)
Unemployed	6 (13.3)	3 (15)	2 (13.3)	1 (10)
Total household income, n (%)				
Less than US $25,000	22 (48.9)	13 (65)	4 (26.7)	5 (50)
US $25,000‐$49,999	19 (42.2)	7 (35)	8 (53.3)	4 (40)
US $50,000 or more	2 (4.4)	—	1 (6.7)	1 (10)
Refused to answer	2 (4.4)	—	2 (13.3)	—

### Iterative Development Process

#### Conceptual Prototypes

Twenty adolescents (mean 13.5, SD 1 year old, 13/20, 65% female, BMI percentile: mean 59.1, SD 26.1) and their parents (mean 43.2, SD 6.2 years old, 18/20, 90% female, BMI: mean 30.1, SD 12.2) participated in this phase. Participants’ responses on the MDPQ-16 showed high mobile device proficiency for both parents and adolescents across all eight dimensions (Multimedia Appendix 5).

In focus groups and individual interviews, parents and adolescents defined a healthy lifestyle as one that includes exercise, healthy eating (eg, fruits and vegetables, limited junk food and sugar), adequate hydration, and sufficient sleep. They rated their health as “average” or “in between,” acknowledging both positive habits and their need for improvement. They described limited use of technology for health purposes, primarily for internet searches related to medical symptoms, recipes, and workouts. Most reported not having any health-related apps installed on their smartphones. When asked about features they liked about either health apps or their favorite apps in general, parents and adolescents emphasized the importance of interactive, social features, such as the ability to communicate with others, and regularly updated content or information. Participants, particularly adolescents, expressed a desire for minimal reading requirements.

**Figure 2.**
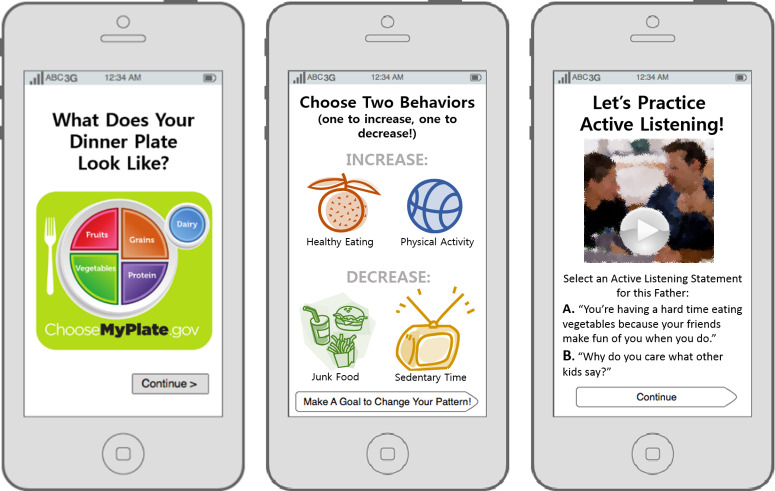
Conceptual prototypes.

Participants viewed conceptual prototypes in their preferred language (English or Spanish) developed by the PI using PowerPoint (Microsoft Corp) to illustrate 3 possible components or features of a family-based digital lifestyle intervention based on the conceptual framework: (1) didactic information on healthy lifestyle behaviors, (2) a family behavior change tool, and (3) a positive parenting tool (Figure 2).

In response to these prototypes, participants suggested that the intervention provide culturally relevant healthy meal recipes, information about nutrition labels, and appropriate portion sizes. They also recommended that we describe the benefits of exercise for adolescents and adults and offer workout routine ideas. Regarding behavior change, participants suggested that we include individualized goal setting with visuals to track their progress, a way for family members to share goals and progress, and reminders and motivational messages. Finally, regarding parenting, parents suggested that we provide strategies to improve family communication, tips on how to deal with difficult situations, and ways to motivate and reward adolescents. They also recommended that we include affordable options for family activities.

#### Paper and Minimally Functional Prototypes

**Figure 3.**
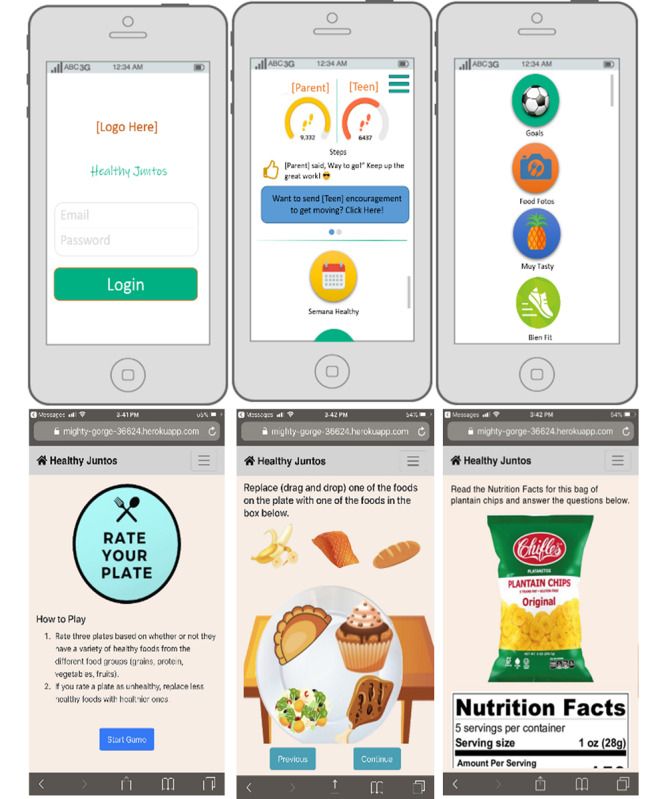
Paper and minimally functional prototypes.

We developed a series of 10 paper prototypes using PowerPoint, and our technology partners (led by author RS) developed minimally functional prototypes to preliminarily assess product usability (Figure 3).

The paper prototypes included representations of different icons and features of the intervention (ie, a sample log-in page, a home page with key icons, and separate pages that corresponded to each icon). Illustrations showed educational tools (ie, didactic health videos, serious games, culturally tailored recipes, and exercise tutorials), behavior skills training tools (ie, step counting, monitoring diet, and physical activity), and parenting and family motivational tools (ie, a parenting podcast, family goal comparison, and family activities). We envisioned that participants would receive weekly didactic content, set and monitor physical activity and healthy eating goals, and engage in weekly family discussions and health challenges. Parents would also have access to a parenting “podcast” to learn skills for positive communication and parenting (eg, active listening). Both parents and adolescents could compare progress, exchange preselected encouraging messages, maintain a food photo journal, participate in weekly games (eg, create your own healthy plate), and learn about the cultures and traditions of different Latin American countries each week. The minimally functional prototypes were of learning activities that aligned with didactic content. These activities included tasks like dragging and dropping healthier food options onto a plate, reading and answering questions about a nutrition label, and timing themselves while doing various physical activities.

Fifteen adolescents (9/15, 60% female; mean 14.2, SD 1.3 years) and their parents (14/15, 93% female; mean 46.3, SD 4.7 years) participated in this phase. Participants suggested improvements related to aesthetics (eg, icon design, image quality) comprehension (eg, simple directions, better feature descriptions), content (eg, removal of certain features), and functionality (eg, different buttons; Table 3). Overall, participants provided mostly positive feedback on the prototypes, highlighting ease of use, functionality, the ability to compare parent and adolescent progress, and enthusiasm for recipes and exercise components. Adolescents were content with the organization of the pages and the color scheme. The intervention prototypes received positive ratings from both parents and adolescents, with USE questionnaire average scores higher than 5 (out of 7) across all four usability dimensions (Multimedia Appendix 5).

**Table 3.** Participants’ feedback on paper/minimally functional prototypes and corresponding modifications.

Feedback	Illustrative quotes	Modifications
Aesthetics Intervention logo should include representation of family, healthy eating, and/or physical activity Replace icon images Change background color	“Well, because it involves family and it involves parents and the kids, that’s why I’m thinking of like the logo of like, you know, you see the silhouette, like the stick figures type deal, with like a bigger one, a smaller one, where you could tell it’s a family.” (Parent 009)	Logo developed with all these features For example, replaced soccer ball icon with progress graph to represent “set your goals” feature Replaced white background with light peach color
Comprehension Lack of clarity about functioning and tasks related to certain features Parents did not understand meaning of “podcast”	“The ‘Podcast,’ is that like people who are in the same boat. Like a community something?” (Parent 006)	Clearly labeled icons, included simple instructions when needed, and developed instructional welcome video to describe all features of intervention Replaced “podcast” with “radio”
Content Lack of clarity about functioning and tasks related to certain features Lukewarm response to photo food diary	“It could also be like. I don’t know, [.] instead of putting photos and types of foods, put the calories, like putting. Today, I ate this many calories.” (Adolescent 005)	Clearly labeled icons, included simple instructions when needed, and developed instructional welcome video to describe all features of intervention Replaced photo food diary feature with simple food log for self-monitoring
Functionality Faulty “drag and drop” feature in minimally functional game prototype Parents did not understand the gallery-style dots indicating the need to swipe to move to next page	“Maybe add ‘linear,’ because they like that, because it kind of tells them: You’re going up and down.” (Parent 006)	Replaced with “click to select” Replaced with a toggle switch that required clicking rather than swiping

#### Fully Functional Prototype

**Figure 4.**
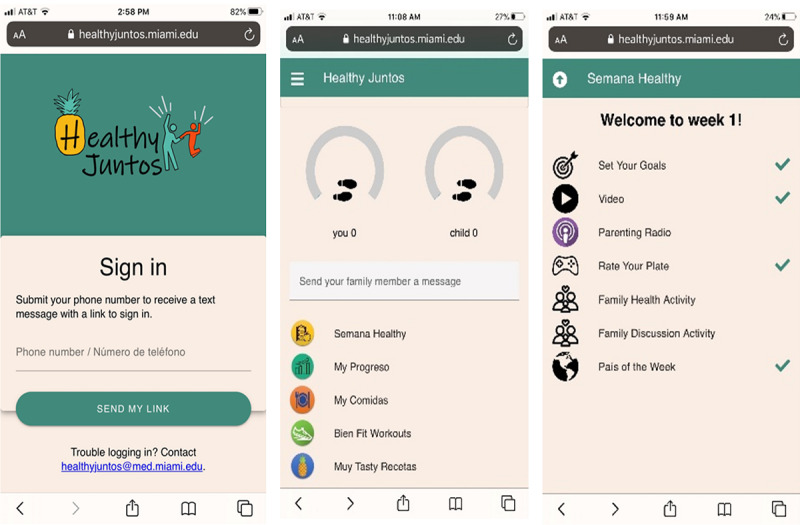
Fully functional prototype version 1.0 (before field trial).

We made significant changes to our prototypes and worked with our software development partners to build out a fully functional prototype (Figure 4) that included eight modules of content and corresponding activities.

We made changes to aesthetics (eg, updated icons and logo), comprehension (eg, enhanced clarity through clearly labeled icons and an instructional welcome video), and content (eg, workout videos to emphasize proper form and incorporated healthy alternatives to typical Hispanic foods). To address issues with the functionality of games, we added clear instructions to each page, replaced the “drag and drop” feature with a simpler “click to select,” and incorporated congratulatory messages upon game completion. We simplified food tracking by requiring participants to simply enter consumed foods, reducing the burden on users. In addition, our team created animated video clips to deliver didactic content, identified publicly available video clips of culturally relevant recipes, produced short exercise suggestion and safety videos, recorded audio clips to deliver positive parenting content, and worked on programming behavior change features (eg, goal setting, self-monitoring, and family support).

As illustrated in Figure 4, the home page gave participants access to content and features through five icons: (1) Semana Healthy (healthy week), (2) My Progresso (my progress), (3) My Comidas (my foods), (4) Bien Fit Workouts (very fit workouts), and (5) Muy Tasty Recetas (very tasty recipes). Although all content was available in English and Spanish (based on participants’ preferred language), the icons on the home page were labeled in Spanglish (ie, combination of English and Spanish) to appeal to adolescents’ biculturalism [47]. The Semana Healthy icon contained all weekly modules (for a total of 8 modules; see Table 4). Within Semana Healthy, participants could complete self-guided weekly tasks that included setting weekly goals for daily steps and healthy meals, watching two to three 2-to-5-minute animated didactic videos, playing a game that reinforced weekly didactic content, and learning about physical activities (eg, salsa dancing) and foods from a featured Spanish-speaking country. Parents additionally had access to one weekly 10‐15 minute “Parenting Radio” episode hosted by the PI that discussed family communication and positive parenting skills. Except for the “Parenting Radio” feature, all content was the same for parents and adolescents. Finally, within Semana Healthy*,* parent-adolescent dyads were prompted to increase healthy interactions and communication through health and discussion activities. For family health activities, participants were prompted to select at least one of three healthy activities (eg, go for a walk together). For family discussion activities, participants discuss 2‐5 open-ended questions. Participants could document their completion of these activities through typing a summary in a text box. The app also included self-monitoring features (“My Progreso,” “My Comidas”) that allowed participants to view their own and their dyad member’s average daily steps (read in from Fitbit) and type in the foods they ate. They could also access a library of 40 workout videos with cues for form and safety created by our team (“Bien Fit Workouts”) as well as links to 46 culturally relevant video recipes from Kiwilimón.com (“Muy Tasty Recetas”).

**Table 4.** Healthy Juntos module content.

Module	Didactic videos	Family health activity	Parenting radio	Family discussion activity
1	What is Healthy Eating? Making the Switch to a Healthy Lifestyle How Much Should I Eat?	Option 1: Cook a healthy meal together. Option 2: Go grocery shopping together and pick two new healthy foods you will try. Option 3: You choose!	Family Communication: Active Listening	How are we doing with our healthy eating as a family? What are we each doing well with our eating habits? What can we improve? How well do we listen to one another when we are communicating?
2	All About Exercise Let’s Move! The Benefits of Exercise Are You Sitting Too Much?	Option 1: Go for a walk together after a meal. Option 2: Do a workout from “Bien Fit” together Option 3: You choose!	Family Communication: Responding	How are we doing with our physical activity as a family? What are we each doing well with our physical activity? What can we improve? How well do we respond to one another when we are communicating?
3	Reading Food Labels Decode the Food Label Crazy Food Cravings	Option 1: Read and discuss the nutrition labels of 2‐3 items in your pantry. Option 2: Make a cooking swap. For example, try using whole wheat pasta instead of white pasta. Option 3: You choose!	Supporting Autonomy and Giving Praise	What are some ways we can support one another with healthy eating? What can we say to encourage each other? What can we do to help each other on hard days? What are some ways we can support one another with physical activity? What can we say to encourage each other? What can we do to help each other on hard days?
4	Cardio 101 Step It Up!	Option 1: Do jumping jacks during breaks while watching your favorite show together. Option 2: Work together to calculate your maximum heart. Do 30 seconds of jumping jacks and take your pulse (see Video 1 this week). Option 3: You choose!	Family Problem Solving	What rules could we come up with as a family to help us improve our healthy lifestyle behaviors? (Come up with 1‐2 rules for your family related to either physical activity, sedentary behavior, or healthy eating) How can we help each other stick to these rules? What happens when we follow the rules? What happens when we don’t?
5	Food is Medicine What Can Food Do For Me? The ABCs of Vitamins	Option 1: Cook a healthy meal together. Option 2: Make a healthy smoothie together. Option 3: You choose!	Monitoring and Managing Peers	Our friends can influence us in many different ways, both positive and negative. How do our friends influence our eating habits? How do our friends influence our physical activity? How can we involve our friends in making healthy choices with us?
6	Resistance Training 101 Strength and Endurance	Option 1: Complete the Fitbit Weekend Warrior challenge. Option 2: Do an active household chore together. Option 3: You choose!	Managing Other Influences on Our Health	How does our extended family influence our eating habits? How does our extended family influence our physical activity? Who in our extended family should we tell about our health goals? What kind of support do we want from them? What (and when) will we tell them?
7	I Can Do That In My Sleep How to Become a Super Sleeper Eating Mindfully Mindful Eating: Practice	Option 1: Try the Healthy Juntos mindful eating activity together. Option 2: Turn your TVs and cell phones off one night! Have a family game night instead. Option 3: You choose!	Managing Parenting and Other Stress	Stress can get in the way of our physical activity and healthy eating habits. What makes you feel stressed? How do you know when you feel stressed? How does stress impact your physical activity and eating habits? What are some things we can do to better handle stress?
8	Flexibility 101 Stretch and Reach for the Stars	Option 1: Start the morning with a healthy breakfast together and 3 yoga poses. Option 2: Do a flexibility workout from “Bien Fit” together. Option 3: You choose!	Staying Healthy and Juntos	What has our family learned from doing Healthy Juntos? What positive changes have we made? How will we maintain or continue to make progress toward our health goals? How will we continue to support one another?

Ten adolescents (mean 13.9, SD 1.3 years old; 5/10, 50% female, BMI percentile: mean 65.3, SD 25.5) and their parents (mean 43.7, SD 5.6 years old; 10/10, 100% female, BMI: mean 25.8, SD 2.5) agreed to participate in this phase. Eight of ten families enrolled in our field trial completed all weekly sessions and postintervention interviews. Results from the USE questionnaire showed scores above 6 (out of 7) for both parents and adolescents across all 4 usability dimensions, indicating high satisfaction with Healthy Juntos and perceiving the intervention as useful and easy to learn and use (Multimedia Appendix 5).

In exit interviews, participants reported a positive experience, with many indicating improved communication and a sense of “feeling closer.” In addition, they reported increased physical activity levels and healthier dietary choices, such as consuming more vegetables and reducing unhealthy food intake. In terms of intervention usage, most parents reported completing the required weekly activities in a single session, while adolescents often fell behind their parents’ pace. The most positively received intervention components were the didactic videos, parenting radio episodes, and the healthy games feature. However, the messaging feature was underused, with participants preferring verbal motivation. In addition, many participants found the progress tracking feature confusing and commented that logging their meals was tedious, as was keying in summaries of their family health and discussion activities. Most did not use the recipes and suggested including easier, more diverse, and “fun” recipes.

Notably, all but one parent expressed interest in having weekly or biweekly human contact to help them overcome barriers and provide feedback. Our experience managing the field trial also indicated the need for more systematic human support. To encourage families to complete sessions, we supplemented automated text reminders with phone calls to parents. Parents used these calls to solicit technical and problem-solving support.

**Figure 5.**
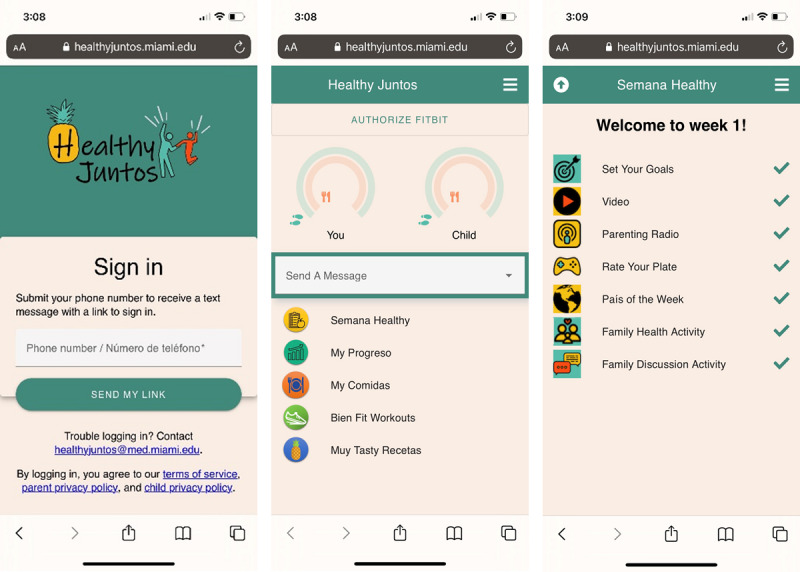
Fully functional prototype version 2.0 (after field trial).

Based on participants’ feedback, we made a final round of modifications to enhance aesthetics, comprehension, content, and functionality in preparation for a pilot randomized controlled trial (Table 5 and Figure 5). We also developed a health coaching protocol for brief weekly video conference consultations with parents.

**Table 5.** Participants’ feedback on fully functional prototype and corresponding modifications.

Feedback	Illustrative quote	Modifications
Aesthetics, Comprehension, Functionality Having to log in to the app prior to each use was a barrier Underused messaging feature; some said they “did not notice it” on the home page Participants did not know to swipe to see the progress bars for healthy meal tracking on the home page Did not like keying in foods on our food log—reported it was tedious Typing summaries of family health and discussion activities was cumbersome	“At first, it [log-in process] made me uncomfortable, but then I got used to it.” (Parent 1001)	Developed simplified log-in procedures Made messaging feature more prominent on the home page—outlined in a box Included a single set of progress bars (that had outer layer for viewing steps and inner layer for viewing meals ratings) Leveraged Fitbit to track meals (to facilitate simpler search for foods); added a self-rating of their logged meals (read in from Fitbit) as healthy, a little healthy, or not healthy to our app Added the option to record a brief audio clip to report on family health and discussion activities
Content Positive feedback on didactic videos, parenting radio episodes, and healthy games feature Did not use the recipes; suggested easier, more diverse, and “fun” recipes	“Since there’s people that like me that are picky, there are times that the breakfast one. I wouldn’t like the options they had, so maybe, yeah, more simple ones will be nice.” (Adolescent 1005)	Audited suggested recipes; replaced “difficult” ones with those that required less ingredients; attempted to include more diverse cuisines based on country of origin

## Discussion

### Principal Findings

Digital interventions hold great promise for delivering family-based lifestyle interventions to Hispanic youth by providing accessible, scalable, and personalized support for sustaining behavior change. The current study informs the development of a digital family-based lifestyle intervention for Hispanic adolescents and parents. We followed the first seven phases of the IDEAS framework to align end user needs and preferences with theoretical constructs and intervention concepts and prototypes. Active involvement of participants in the development process was crucial for refining the intervention and ensuring cultural relevance.

Previous studies have used co-design processes to develop digital lifestyle interventions for Hispanic youth [48,49]. These collaborative approaches provided crucial insights for generating content that is both engaging and relevant. By involving Hispanic adolescents and their parents in the iterative development process of Healthy Juntos, we collected valuable data that directly informed the content, design, and functionality of our intervention. We were responsive to participants’ feedback and Hispanic cultural values through including culturally relevant recipes, encouraging exercise using everyday household items, and producing content (eg, parenting radio) that covers topics Hispanic parents are likely to encounter raising adolescents in the United States.

Based on the participants’ usage of the fully functional prototype and their qualitative feedback, we identified several actionable changes that have already been implemented. These changes include simplifying the log-in process, increasing the frequency of text reminders with standard motivational messaging, and reducing the reporting burden for family health and discussion activities (through the addition of an audio recording option). Because participants found the manual logging of diet to be tedious, we also enhanced integration with the Fitbit app to simplify meal tracking. In light of documented challenges with self-monitoring lifestyle behaviors among adolescents [50,51], this change was intended to improve adherence to self-monitoring, a key determinant of successful health behavior change [52,53].

Another significant finding with direct implications for our future pilot RCT was the high demand for human support. In response, we developed a protocol for weekly health coaching sessions to foster supportive accountability through personalismo (personal connection) and confianza (trust), which have been shown to enhance the engagement and retention of Hispanic study participants [54]. This need for human support in digital health mirrors findings from other co-design studies in this population. For instance, Spierling Bagsic et al [55] evaluated Dulce Digital-Me, an adaptive mobile health (mHealth) intervention for Hispanics with diabetes, comparing personalized feedback via automated text messages to health coach phone calls. Participants in the health coach arm were more engaged in the intervention [55] and showed greater improvements in diabetes self-management behaviors (eg, healthy diet) than those in the automated text message arm [56]. We hypothesize that incorporating health coaching sessions as part of Healthy Juntos will enhance engagement and retention of Hispanic participants.

A pilot RCT (n=50) will evaluate the feasibility, acceptability, and preliminary effects of the refined Healthy Juntos intervention on improving physical activity and diet quality among Hispanic adolescents and their parents (IDEAS phase 8). We anticipate that our intervention will be both feasible and acceptable among our target population. Furthermore, we hypothesize that participants in the Healthy Juntos arm will show promising effects compared to the control group in increasing MVPA, reducing sedentary behavior, and improving diet quality among adolescents at postintervention.

### Limitations and Strengths

The small sample size may limit the generalizability of the findings to a broader population. That said, we followed a systematic framework to guide our intervention development process, providing a model that other researchers can replicate in different contexts and populations. In addition, we acknowledge that our findings might be affected by social desirability bias, in which participants could overstate positive feedback or understate negative aspects of the prototypes to please the researchers. However, by actively involving end users (ie, Hispanic adolescents and their parents) throughout the development stages, we ensured that the intervention addresses their specific needs and preferences. A user-centered design approach may increase the relevance and acceptability of the intervention, facilitate iterative improvements, and enable the timely identification and resolution of potential issues.

Regarding reliance on technology, although Hispanic adolescents tend to use their phones more than other groups, this does not guarantee ongoing engagement with a health app. Initial interest might fade, leading to app abandonment. Future steps include testing the refined intervention in a pilot RCT and soliciting additional qualitative feedback from participants to make further improvements.

### Conclusions

The iterative process of developing Healthy Juntos through qualitative feedback and a field trial was crucial for its refinement. Hispanic families actively contributed to the development of the intervention and found it easy to learn and useful. Hispanic parents were especially enthusiastic about the parenting radio content and opportunities to engage with their adolescents. Adolescents reported enthusiasm for videos and games. Moving forward, we will rigorously evaluate its feasibility, acceptability, and efficacy through a pilot study and a fully powered randomized controlled trial.

## Supplementary material

10.2196/73848 Multimedia Appendix 1 Participant flow diagram.

10.2196/73848 Multimedia Appendix 2 Conceptual prototypes question guide.

10.2196/73848 Multimedia Appendix 3 Paper and minimally functional prototypes question guide.

10.2196/73848 Multimedia Appendix 4 Fully functional prototype question guide.

10.2196/73848 Multimedia Appendix 5 Participants’ mobile device proficiency and usability.

## Abbreviations

HEI: Healthy Eating Index
IDEAS: Integrate, Design, Assess, and Share
MDPQ-16: Mobile Device Proficiency Questionnaire
MVPA: moderate-to-vigorous physical activity
PI: principal investigator
RCT: randomized controlled trial
USE: Usefulness, Satisfaction, and Ease of use
