# Supplementary figures and images for: Family-Based Digital Lifestyle Intervention for Hispanic Adolescents and Their Parents: Iterative Co-Design and Development Study
Source: JMIR Form Res. 2026 Feb 5;10:e73848. doi: 10.2196/73848 (PMC12875426; doi:10.2196/73848)

Appendix 1. Participant Flow Diagram


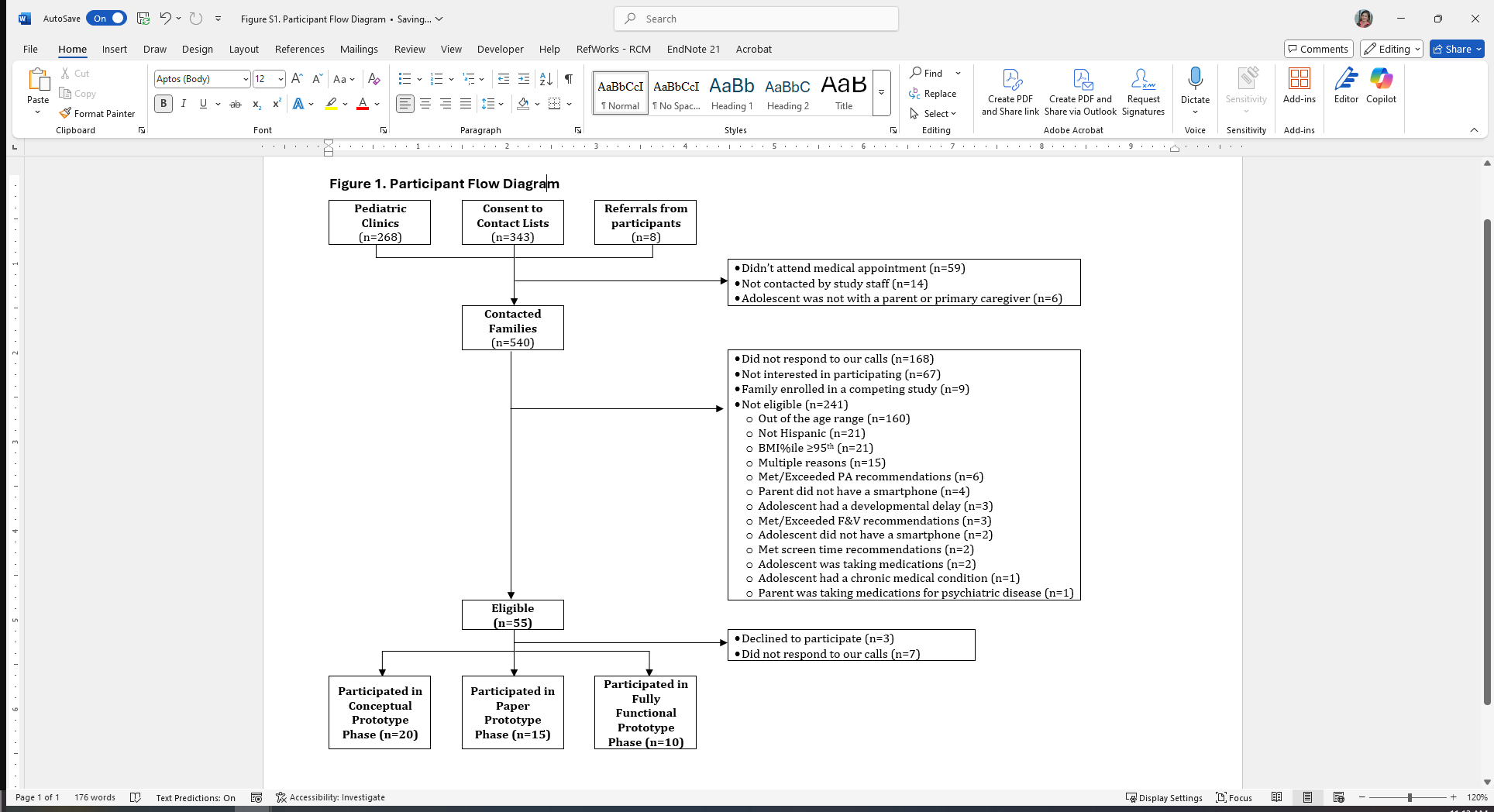

Supplement: Multimedia Appendix 1 [file formative-v10-e73848-s001.docx]
