# Supplementary material for: Family-Based Digital Lifestyle Intervention for Hispanic Adolescents and Their Parents: Iterative Co-Design and Development Study
Source: JMIR Form Res. 2026 Feb 5;10:e73848. doi: 10.2196/73848 (PMC12875426; doi:10.2196/73848)
Supplement: Multimedia Appendix 2 [file formative-v10-e73848-s002.docx]

Appendix 2. Conceptual Prototypes Question Guide

**Questions:**

Before we talk more about technology and how it might relate to health, we are going to spend some time talking about health in general.

1. What do you consider a healthy lifestyle?
2. How healthy do you think you are? How do you know?
3. How (if at all) have you tried to change your healthy lifestyle behaviors (physical activity and healthy diet)?
   1. *Probe:* What has worked?
   2. *Probe:* What has not worked?
4. How (if at all) are you healthy together with your parent/adolescent?
   1. *Probe*: What types of healthy activities do you do with your parent/adolescent?
   2. *Probe*: If you have conversations about your health, what do you discuss with your parent/adolescent?

The next set of questions relate to health and technology.

1. How (if at all) have or do you use technology (computers, tablets, mobile phones/smartphones) for your health/healthcare?
   1. *Probe*: How (if at all) have or do you use technology to increase your physical activity?
   2. *Probe*: How (if at all) have or do you use technology to improve your healthy eating?
   3. *Probe:* [If participants are reporting they do not use technology to improve health behaviors] What would motivate you to use technology to increase your physical activity or improve your healthy eating?
2. What (if any) health applications (apps) do you have on your smartphones?
   1. *Probe*: Why did you download these apps?
   2. *Probe*: What features did you like best about these apps?
   3. *Probe*: What features did you dislike about these apps?
   4. *Probe*: How long did you use these apps? Why did you stop using them?What features did you like best about these apps?
      es are used on a daily basis]. es) on a daily basis. rticipando en el estudio

Now we would like to get your feedback on an online program we are in the process of developing to improve healthy lifestyle behaviors including physical activity and nutrition in Hispanic adolescents and their primary caregivers. Our idea is that this program would be accessed through your smartphone but you can also access it from a computer. We are thinking that the program will be 8 weeks long and will have three key tools: 1) a health education/information tool, 2) a behavior change tool, and 3) a positive parenting tool. We are going to show you an image of each of these tools and would like to know what you think of them. You may take a minute or two to write down any thoughts you have about them before we ask you questions about them.

The first tool is the health education/information tool [*show tool on screen or on paper*]. Our idea is that this tool would provide you with a different lesson on physical activity and a healthy diet each week.

1. What (if anything) would make you want to use this tool?
   1. *Probe*: What do you recommend we include?
   2. *Probe*: What types of lessons/info would you want to learn?
   3. *Probe*: What would make it fun or enjoyable for you to use this tool?

The second tool is a health behavior change tool [*show tool on screen or on paper*]. Our idea is that this tool would be used to help you set goals every week for improving your healthy lifestyle behaviors, track how you are doing with those goals, and allow your parent/adolescent to see your goals.

1. What (if anything )would make you want to use this tool?
   1. *Probe*: What do you recommend we include?
   2. *Probe*: What features would motivate you to use this tool to improve your family’s healthy lifestyle behaviors?

The third tool is a health behavior change tool [*show tool on screen or on paper*]. Our idea is that this tool would be used by parents only to help them support your family in getting healthy by learning a new family support strategy, like listening, each week.

1. For parents only: What (if anything) would make you want to use this tool?
   1. *Probe*: What do you recommend we include?
   2. *Probe*: What types of parenting issues would you want tips about?

For adolescents only: What do you think your parents should know to help you be healthy?

1. Now that you have seen all three tools, we would like to know what other ideas or thoughts you have for us as we develop this program. What else (other tools or features) should we consider including?
2. Have we left anything out or not talked about something that you feel is important to our discussion today? Please explain.
