# Supplementary material for: Family-Based Digital Lifestyle Intervention for Hispanic Adolescents and Their Parents: Iterative Co-Design and Development Study
Source: JMIR Form Res. 2026 Feb 5;10:e73848. doi: 10.2196/73848 (PMC12875426; doi:10.2196/73848)
Supplement: Multimedia Appendix 3 [file formative-v10-e73848-s003.docx]

Appendix 3. Paper and Minimally Functional Prototypes Question Guide

Now we are going to show you some mock-ups of the program.

1. **Mock up #1:** This is the login page of the program. It includes the name of the program “Healthy Juntos.” We have not yet designed our logo – that picture is there to represent where the logo will go.
   1. What images come to mind when you hear “Healthy Juntos”? What ideas do you have for our logo?
2. **Mock up #2:** This is the home page of the program.
   1. What would you do on this page (e.g., scroll, click, etc)? [*Allow participants to interact with the page and encourage them to talk aloud as they do. Gently prompt them by saying “keep talking” if they are silent. Then proceed with the following question probes to encourage continued talking*.]
   2. What do you think of the layout of this page?
      1. What information on this page (if anything) looks interesting to you? Why?
      2. What (if anything) on this page is difficult to understand/confusing? Why? What would make it easier to understand?
   3. Where would you click? Why did you decide to click on [*insert what was clicked*]?
   4. What do you think each icon represents? What do you think happens when you click it?
   5. [*If participant doesn’t know to swipe at the top*] What do you think these dots represent? [*Show them what screen comes up after swiping*]
3. **Mock up #3:** This display shows what you see when you click the icon “Semana Healthy.”
   1. What would you do on this page (e.g., scroll, click, etc)? [*Allow participants to interact with the page and encourage them to talk aloud as they do. Gently prompt them by saying “keep talking” if they are silent. Then proceed with the following question probes to encourage continued talking*.]
   2. What do you think each of these images [*pointing to learning map*] represents? What do you think happens when you click it?
4. **Mock up #4:** This display shows what you see when you click the icon “Goals.”
   1. What would you do on this page (e.g., scroll, click, etc)? [*Allow participants to interact with the page and encourage them to talk aloud as they do. Gently prompt them by saying “keep talking” if they are silent. Then proceed with the following question probes to encourage continued talking*.]
   2. What do you think these graphs represent?
5. **Mock up #5:** This display shows what you see when you click the icon “Food Fotos.”
   1. What would you do on this page (e.g., scroll, click, etc)? [*Allow participants to interact with the page and encourage them to talk aloud as they do. Gently prompt them by saying “keep talking” if they are silent. Then proceed with the following question probes to encourage continued talking*.]
   2. How useful do you find this feature? How often would you use it?
   3. If not with pictures, how else would you like to keep track of foods you and your family members have eaten?
6. **Mock up #6:** These displays show what you see when you click either “Muy Tasty” or “Bien Fit.”
   1. What would you do on these pages (e.g., scroll, click, etc)? [*Allow participants to interact with the page and encourage them to talk aloud as they do. Gently prompt them by saying “keep talking” if they are silent. Then proceed with the following question probes to encourage continued talking*.]
   2. How useful/valuable do you find these features? How often would you use them?
   3. How open are you to trying healthier alternatives to typical Latin foods?
   4. How important is having exercise/workout videos available to you?
7. **Games**: These displays show you some games we have in the program.
   1. How would you play this game?
   2. How did you like this game?
   3. How (if at all) would you recommend that we improve this game (e.g., design, colors, functionality)?
   4. What (if anything) did you learn from playing this game?
8. **General Questions:** I’m now going to ask you some general questions about your overall experience with the program.
   1. What did you like most/least about the mock website?
   2. How could it be improved?
   3. How likely are you to use a website like this?
   4. What do you think about the colors and overall design?
   5. Have we left anything out or not talked about something that you feel is important to our interview today? Please explain.
