# Supplementary material for: Family-Based Digital Lifestyle Intervention for Hispanic Adolescents and Their Parents: Iterative Co-Design and Development Study
Source: JMIR Form Res. 2026 Feb 5;10:e73848. doi: 10.2196/73848 (PMC12875426; doi:10.2196/73848)
Supplement: Multimedia Appendix 4 [file formative-v10-e73848-s004.docx]

Appendix 4. Fully Functional Prototype/Field Trial Question Guide

Now that you have completed the entire program, I’d like to ask you more general questions about your overall experience in the program.

1. Describe your overall experience using the program.
   1. Probe: What did you like most/least about the program?
   2. Probe: What was your favorite/least favorite part about the program?
2. What goals did you have for using the program?
3. How (if at all) did the program help you meet these goals?
4. How (if at all) did the program impact the health behaviors of your family?
5. How (if at all) did the program impact your relationship with your adolescent/parent?
6. What improvements would you make?
7. Did you have any technical issues using the program during these past eight weeks?
   1. Probe: If yes, what were these issues?
8. How helpful do you think it would be if a member of our team called you during the eight-week program to check in on your family’s progress?
   1. Probe: How often would you like these calls to be?
