# Supplementary material for: Family-Based Digital Lifestyle Intervention for Hispanic Adolescents and Their Parents: Iterative Co-Design and Development Study
Source: JMIR Form Res. 2026 Feb 5;10:e73848. doi: 10.2196/73848 (PMC12875426; doi:10.2196/73848)
Supplement: Multimedia Appendix 5 [file formative-v10-e73848-s005.docx]

Appendix 5. Participants’ Mobile Device Proficiency and Usability

**Table 1.** Mobile Device Proficiency Questionnaire (MDPQ-16)

|  | **Adolescents (n=20)** | | **Parents (n=20)** | |
| --- | --- | --- | --- | --- |
|  | **Mean** | **SD** | **Mean** | **SD** |
| **Mobile Device Basics** | 4.65 | 0.56 | 4.55 | 0.52 |
| **Communication** | 4.20 | 1.17 | 4.60 | 0.58 |
| **Data and File Storage** | 4.00 | 1.00 | 4.03 | 1.04 |
| **Internet** | 4.55 | 0.56 | 4.48 | 0.68 |
| **Calendar** | 4.28 | 1.02 | 3.85 | 1.57 |
| **Entertainment** | 4.93 | 0.18 | 4.38 | 0.74 |
| **Privacy** | 4.58 | 0.63 | 4.15 | 1.04 |
| **Troubleshooting** | 4.98 | 0.11 | 4.20 | 1.14 |
| **Total Score** | 36.15 | 2.89 | 34.23 | 6.42 |

*Note.* Items on the MDPQ-16 range from 1 (*never tried at all*) to 5 (*very easily*). The measure yields eight subscales and one total score, with higher scores reflecting greater mobile device proficiency.

**Table 2.** Usefulness, Satisfaction, and Ease of use (USE) questionnaire

|  | **Paper and Minimally Functional Prototype** | | | | **Fully Functional Prototype** | | | |
| --- | --- | --- | --- | --- | --- | --- | --- | --- |
|  | **Adolescents (n=15)** | | **Parents (n=15)** | | **Adolescents (n=8)** | | **Parents (n=8)** | |
|  | **Mean** | **SD** | **Mean** | **SD** | **Mean** | **SD** | **Mean** | **SD** |
| **Usefulness** | 5.89 | 0.90 | 6.28 | 0.79 | 5.89 | 0.87 | 6.13 | 0.94 |
| **Ease of Use** | 5.96 | 0.77 | 6.50 | 0.69 | 6.25 | 0.71 | 6.13 | 1.33 |
| **Ease of Learn** | 6.53 | 0.65 | 6.78 | 0.57 | 6.78 | 0.45 | 6.19 | 1.07 |
| **Satisfaction** | 5.95 | 0.91 | 6.20 | 1.11 | 6.02 | 0.68 | 6.04 | 1.23 |
| **Total Score** | 6.09 | 0.73 | 6.44 | 0.68 | 6.22 | 0.53 | 6.12 | 1.11 |

*Note.* Items on the USE range from 1 (*strongly disagree*) to 7 (*strongly agree*). The measure yields four subscales and one total score, with higher scores reflecting greater usability of the program.
